# The Relationship Between Periodontal Disease and Public Health: A Population-Based Study

**DOI:** 10.5539/gjhs.v8n7p110

**Published:** 2015-11-18

**Authors:** Somaye Ansari Moghadam, Zohre Abdollahi, Sirous Risbaf Fakour, Alireza Ansari Moghaddam, Farin Kiany, Nourmohamad Damani

**Affiliations:** 1 Oral and Dental Disease Research Center, Zahedan University of Medical Sciences, Zahedan, Iran; 2 Zahedan University of Medical Sciences, Kerman, Iran; 3 Health Promotion Research Center, Zahedan University of Medical Sciences, Zahedan, Iran; 4 Prevention of Oral and Dental Disease Research Center, School of Dentistry, Shiraz University of Medical Sciences, Shiraz, Iran; 5 Zahedan University of Medical Sciences, Zahedan, Iran

**Keywords:** periodontal disease, public health, tooth loss

## Abstract

**Background:**

Periodontal diseases, such as periodontitis, are considered the main cause of tooth loss in the elderly. The present study is aimed to determine the relationship between periodontal condition and quality of life. Quality of life consists of a range of people’s objective needs related to the self-perception of well-being.

**Methods:**

This study was done from January 2014 to June 2015 in a healthcare clinic in Zahedan, southeast Iran. Using the random sampling method, the researchers enrolled 700 individuals over 35 years of age. The participants initially completed a demographic questionnaire consisting of data, such as age, sex, educational level, and smoking habit. Then, the periodontal chart was completed. Moreover, patients, based on the number of their natural teeth, were divided into two groups (≥10 teeth in both maxillary and mandible arches and <10 teeth in at least one arch). The body mass index (BMI) was also measured. To assess the participants’ general health, the WHO’s quality of life questionnaire (WHOQOL-BREF) was used.

**Results:**

Of the 700 enrolled individuals, 53.3% were womenand 47.7% were men. Moreover, most of the participants (63.71%) had BMI of less than 25 and 68% did not smoke. We found that as the people’s periodontal status deteriorated, their quality of life also declined and the total mean score in all four health domains decreased (P<0.001). Moreover, people with more than 10 teeth in both arches scored higher with respect to life quality than those with less than 10 teeth in at least one arch (P<0.001).

**Conclusion:**

This studyindicates a decrease in the general quality of life in patients with periodontal disease. The authors suggest performing studies with larger sample sizes andcohort studies for more reliable results.

## 1. Introduction

Periodontitis isthe main cause of tooth loss in the elderly. Edentulism is considered to be the final stage of periodontal disease. Periodontal diseases and their complications, such as edentulism, are the common and important problems in the community. It is noticed thatperiodontal diseasesalter individuals’ life style, health status, and social roles, all of which affecting the overall quality of life (Zhang, Wisniewski, & Bauer, 2006). Quality of life is proportionate to meetingpeople’s objective needs that are related to the self-perception of well-being. A desired quality of life is not equivalent to a disease-free life; rather, it means having a feeling of well-being in various aspects, i.e. in mental, social, functional and psychological aspects (World Health Organization quality of life assessment group [WHOQOL Group], 1995). The World Health Organization (WHO) defines the quality of life as people’s perception of their lives and the cultural structure. It also evaluates the system that they live in. This system is related to the goals, expectations, and standards of people (WHOQOL Group, 1995; Namjoshi & Baues Ching, 2001).

Quality of life is evaluated in four different domains: physical, social, psychological, and environmental health. The physical health domain includes the ability to move, perform daily activities, and the capacity to work. And while social health involves personal relationships, social support, and sex life, the psychological health domain deals with perceptions about appearance, self-esteem, thoughts, learning, memory, and concentration. Finally, the environmental health domain focuses on financial resources, physical security, health and social care, physical environment of residence, recreational facilities, as well as air and noise pollution (Sexana, Carlson, & Billington, 2001).

In this study, considering the participants’number of natural teeth and the periodontal condition, the authors aimed to determine and compare the periodontitis patients’ quality of life with that of periodontally healthy individuals.

## 2. Materials and Methods

This study was carriedout in a healthcare clinic in Zahedan, southeast Iran, from January 2014 to June 2015. The number of the participants comprised 700 individuals over 35 years of age, using the random sampling method. Informed consent was obtained from the participants. This study was approved by the Ethics Committee of Zahedan University of Medical Sciences (Code: 1166).

The participants initially completed a demographic data questionnaire, regarding age, sex, educational level and the presence or absence of smoking habit. Then, the periodontal chart was filled using a Williams probe (accuracy of 1 mm) and the participantswere accordingly allocated to three groups (healthy, gingivitis, and periodontitis). All the participants wereexamined for clinical attachment loss (CAL), recession (Rec), probing depth (PD) in 6sites (mesiobuccal, buccal, distobuccal, mesiolingual, lingual, distolingual) on each tooth. Also bleeding index Lenox and plaque index Ol’eary were performed. Gingival index Loe & Sillness and calculus index Russelwere also assessed for the detection of level of gingival inflammation and calculus accumulation. All the examinations were performed by a single operator. Healthy group participants had no signs of gingival inflammation and no evidence of calculus. Bleeding index was less than 10% and plaqueindex was less than 20% in all participants of the healthy group. None of the people in this group neededperiodontaltreatment. Gingivitis patients had no recession and clinical attachment loss (CAL), but had bleeding on probing more than 20%. Those with the following criteriawas enrolledaspatients withchronicperiodontitis: amount of destruction consistent with local factors; sub-gingival calculus; slow-to-moderate rate of progression; clinical attachment loss ≥ 5mm. However, those with aggressive periodontitis, such as familial history of aggressive disease, absence of large accumulation of plaque and calculus, and rapid rate of disease progression, were excluded from the study.

Moreover, depending upon their number of natural teeth (≥10 teeth in both arches and <10 teeth in at least one arch), the participantswere divided into two groups. Finally their body mass index (BMI) was measured, dividing them into two groups: BMI<25, BMI>= 25.

To assess the participants’ general health, we used the WHO’s life quality questionnaire (WHOQOL-BREF), designed to measure the individual’s quality of life during the past two weeks. This questionnaire containsitems relating to the four health domains mentioned earlier. The scores range between 4–20, the higher scores indicating a higher level of life quality (WHOQOL Group, 1995).

Data were analyzed using SPSS software, version 20. T test, linear regression, and ANOVA were used as appropriated.

## 3. Results

Since, the main purpose of this study, was to determine the average of 4 health domains, therefore considering α=0.05, SD= 2, d= 0.2, design effect=1.8, a sample size of 691 was estimated. Accordingly, a total of 700 subjects were enrolled. N= Z^2^ S^2^/d^2^

The demographic characteristics of the participants are shown in Table 1. Of the 700 included individuals, 53.3% were women. Moreover, most of the participants (63.71%) had a BMI of less than 25 and 68% did not smoke. The number of teeth was positively correlated with the quality of life (Table 2). Concerningthe quality of the life, participantswith more than 10 teeth in two maxillary and mandibular arches scored higher than those with less than 10 teeth in at least one arch (P<0.001). Table 3 showsthe health domains scores interms of periodontal status. It was observed that the more the individuals’ periodontal status deteriorated, the lowerwas their quality of life as well as their total mean score in all four domains (P<0.001).

**Table 1:** Frequency (%) of some of the participants’ demographic variables

Variables		Frequency (%)
**Sex**	Women	327 (53.30%)
	Men	373 (46.70%)
**Educational Level**	Illiterate	166 (23.71%)
	<6 years	122 (17.43%)
	6-8 years	150 (21.43%)
	9-12 years	155 (22.14%)
	>12 years	107 (15.29%)
**Body Mass Index**	<25	446 (63.71%)
	≥25	264 (36.29%)
**Smoking**	Yes	234 (32%)
	No	476 (68%)

**Table 2:** Comparison of Health Domain Scores interms of tooth loss

Health Domain	≥lo teeth in both Arches	<lo teeth in one arch at least	p-value*
	
	Mean± SD	Mean± SD	
**Physical**	14.67±1.99	13.11±2.28	<0.001
**Enviromental**	13.80±1.98	12.41±2.05	<0.001
**Social**	13.87±2.19	12.87±2.34	<0.001
**Psychological**	13.40±1.85	12.30±1.86	<0.001

* p-value obtained from T-test.

**Table 3:** Comparison of Health Domain Scores interms of periodontal status

Health Domain	Healthy Group	Gingivitis Group	Periodontitis Group	Total	*p-value
		
	Mean ±SD	Mean ±SD	Mean ±SD		
Physical	15.63±1.93	14.71±1.77	13.47±2.16	14.30±2.17	<0.001
Environmental	14.82±1.85	13.66±1.81	12.76±2.03	13.46±2.08	<0.001
Social	14.28±2.02	13.95±2.06	13.14±2.39	13.62±2.27	<0.001
Phychological	14.24±1.78	13.36±1.75	12.50±1.80	13.12±1.90	<0.001

* p-value obtained from ANOVA.

Moreover, as shown in Table 4, 23% of the variance in the physical health domain could be predicted by age, periodontal condition, educational level, sex, and smoking (adjusted r^2^=0.23); so that with a one-year increase in age, the physical health score decreased by 0.07. Moreover, with an increase in educational level, the physical health score increased by 0.16. The physical health score of people with gingivitis, compared to the healthy individuals, decreased by 0.53. On the other hand, women scored 0.64 less than men in the physical health domain and smokers, compared to non-smokers, had a 0.22 decrease in the physical health score (Table 4).

**Table 4:** Predictors of Health Domains in multiple regression models

Health Domain	Unstandardized Coefficients	Standardized Coefficients	std.Error		Sig
Physical health Predictors					
Constant		16.65	0.83	23.71	<0.001
Age	-0.07	0.01	-0.23	-5.06	<0.001
Periodontal status	-0.53	0.13	-0.19	-4.21	<0.001
Education	0.16	0.06	0.10	2.57	0.01
Gender	0.64	0.19	0.15	3.32	0.001
Smoking	-0.22	0.09	-0.11	-2.50	0.01

Environmental Health Predictors					
Constant		13.28	0.51	26.01	<0.001
Periodontal status	-0.68	0.11	-0.25	-6.40	<0.001
Education	0.34	0.06	0.22	5.98	<0.001
Tooth Less	0.43	0.19	0.09	2.27	0.02

Social Health Predictors					
Constant		16.71	1.01	16.59	<0.001
Education	0.34	0.07	0.21	4.98	<0.001
Age	-0.04	0.01	-0.13	-2.93	0.003
Gender	-0.38	0.17	-0.08	-2.24	0.03

Psychological Health Predictors					
Constant		14.48	0.42	34.17	<0.001
Periodontal status	-0.71	0.09	-0.29	-7.57	<0.001
Education	0.29	0.05	0.21	5.49	<0.001
Gender	-0.38	0.14	-0.1	-2.77	0.006

In the environmental health domain, 20% of the variances in health scores could be predicted by periodontal condition, educational level, and number of teeth (r^2^=0.2) (Table 4). In other words, compared to periodontally healthy individuals, people with gingivitis gained 0.68 lowerscores. And, in people with periodontitis, compared to those with gingivitis, the environmental health score decreased by 0.68. Furthermore, the scores of this domain were positively affected by the educational level (0.34) and the number of teeth (0.43).

Regression analysis showed that 12% of the variance in the social health domain could be predicted by educational level, age, and sex (r^2^=0.12). The social heath score increased by 0.34 as the educational level increased. Moreover, with a one-year increase in age, the social health score decreased by 0.04. The social health score of women was less than that of men by 0.38.

Finally, 18% of the variance in psychological health could be predicted by periodontal status, educational level, and sex (r^2^=0.18). As the periodontal condition deteriorated, the score of this domain decreased by 0.71 but increased by 0.29 as the educational level increased. Furthermore, the psychological health score of women was lower than that of men by 0.38.

## 4. Discussion

Generally speaking, there is a mutual relationship between disease and quality of life. Physical disorders and symptoms directly affect all aspects of the quality of life. As the patients with chronic diseases, compared with healthy individuals, may have a lower life quality, the primary aim of treating chronic diseases is to enhance the patients’ life quality by reducing the impact of the diseases (Smetzer & Bare, 2004; Crakowski, 1999). During the past 20 years, interest in enhancing and evaluating the quality of life of the patients with chronic diseases has increased and enhancing the daily function and quality of life of such patients has become the main aim of healthcare providers (Hofer, Benzer, Schussler, Vonsteinbuchel, & Oldridge, 2003). In the present study, a relationship between periodontal diseases and quality of life in the four related domains was found (P<0.001), using the WHOQOL-BREF. To the best knowledge of the researchers, no similar study has been performed with a similar methodology. All previous studies have focused on oral health related quality of life. Hence, it is difficult to compare the findings of this study with those found in the related literature. However, it is worth mentioning that our findings are consistent with the findings of the study carried out on 85 patients with chronic moderate to severe periodontitis. Teeuw used the Oral Health Impact Profile-NL49 and found a negative association between periodontal disease and quality of life. Moreover, they found that patients with severe periodontitis had lower quality of life compared with those with moderate periodontitis (Teeuw et al., 2011).

Other studies assessing the relation between oral health and quality of life have also found decreased levels of quality of life in periodontitis patients (Cunha-Cruz, Hujoel, & Kressin, 2007; Durhan, Fraser, Mc Cracken, Stone, & John, 2013).

In another study using the Health Profile Impact (OHIP-14), the researchers found an association between periodontal disease treatment and quality of life independent from social and environmental factors. In the mentioned study, the researchers, having assessedthe effect of periodontal treatment 6-8 weeks after treatment and compared it with the baseline, found that treatment was most effective when pocket depth was more than 7 mm (Bernable & Maarcenes, 2010).

We, consistent with the previous reports, found that the quality of life was negatively related to age (Hou, Chui, Eckert, Oldridge, Murray, & Bennett, 2004; Parajou et al., 2004). Moreover, women had lower quality of life compared with men. This can be attributed to the social environment. In any case, this needs to be further assessed in future studies. We also found a positive association between educational status and quality of life. That is to say, higher education creates higher awareness which could, in turn, change the individual’s attitude towards health and disease.

Although oral healthrelated life quality cannot depict the general quality of life in patients, it inevitably affects the patients’ general quality of life. We found an increased quality of life level in patients with higher number of teeth. Although previous studies were done on the relation between the number of teeth and the oral health related quality of life, the results were controversial (Cunha-Cruz et al., 2007; Steele et al., 2004; Mack et al., 2005). Cunha-Cruz and colleagues, e.g., found that the number of teeth losswas a clinical characteristic of patients with periodontal disease, related to lower oral health related quality of life. According to their study, the loss of 25% ofteeth did not affect the patients’ life quality. And if they lose 25-33% of their teeth, their quality of life is significantly affected. However, if they lose more than 33% of their teeth, their quality of life is not affected, showing no linear relationship between the number of teeth and oral health related quality of life (Cunha-Cruz et al., 2007).

In a meta-analysis on tooth loss and oral health related quality of life, Gerritsenet al. found that tooth loss affected the quality of life and this relationship was independent from the type of instrument used to measure oral health related quality of life (Gerrittsen, Allen, Witter, Bronkhorst, & Gruegers, 2010).

The present study was a cross-sectional study and could not prove any cause and effect relationship. Hence, the performance of cohort studies is suggested.

## 5. Conclusion

Considering the results of this study, indicating a decreased general quality of life in patients with periodontal disease and lack of previous studies in this regard, the authors suggest that the prospective researchers replicate the study with larger sample sizes, such as cohort studies.
